# Isomer-specific effects of CLA on gene expression in human adipose tissue depending on PPARγ2 P12A polymorphism: a double blind, randomized, controlled cross-over study

**DOI:** 10.1186/1476-511X-8-35

**Published:** 2009-08-18

**Authors:** J Herrmann, D Rubin, R Häsler, U Helwig, M Pfeuffer, A Auinger, C Laue, P Winkler, S Schreiber, D Bell, J Schrezenmeir

**Affiliations:** 1 Max Rubner-Institut, Federal Research Institute of Nutrition and Food, Department of Physiology and Biochemistry of Nutrition, Karlsruhe and Kiel, Germany; 2 UKSH Kiel, First Department of Medicine, Kiel, Germany; 3 UKSH Kiel, Institute for Clinical Molecular Biology, Kiel, Germany; 4 Center of Clinical Research, Tecura GmbH Medizin & Biotechnik, Kiel, Germany; 5 Nutrition & Health - Global R & D, Cognis GmbH, Monheim, Germany

## Abstract

**Background:**

Peroxisome proliferator-activated receptor (PPAR)γ is a key regulator in adipose tissue. The rare variant Pro12Ala of PPARγ2 is associated with a decreased risk of insulin resistance. Being dietary PPARγ ligands, conjugated linoleic acids (CLAs) received considerable attention because of their effects on body composition, cancer, atherosclerosis, diabetes, obesity and inflammation, although some effects were only demonstrated in animal trials and the results in human studies were not always consistent. In the present study effects of CLA supplementation on genome wide gene expression in adipose tissue biopsies from 11 Ala12Ala and 23 Pro12Pro men were investigated. Subjects underwent four intervention periods (4 wk) in a randomized double blind cross-over design receiving 4.25 g/d of either cis-9, trans-11 CLA, trans-10,cis-12 CLA, 1:1 mixture of both isomers or a reference linoleic acid oil preparation. After each intervention biopsies were taken, whole genome expression microarrays were applied, and genes of interest were verified by realtime PCR.

**Results:**

The following genes of lipid metabolism were regulated by CLA: LDLR, FASN, SCD, FADS1 and UCP2 were induced, while ABCA1, CD36 and CA3 were repressed. Transcription factors PPARγ, NFAT5, CREB5 and EBF1, the adipokine NAMPT, members of the insulin signaling cascade SORBS1 and IGF1 and IL6ST were repressed, while the adipokine THBS1 and GLUT4 involved in insulin signaling were induced. Compared to trans-10,cis-12 CLA and the CLA mixture the cis-9, trans-11 CLA isomer exerted weaker effects. Only CD36 (-1.2 fold) and THBS1 (1.5 fold) were regulated. The CLA effect on expression of PPARγ and leptin genes depends on the PPARγ2 genotype.

**Conclusion:**

The data suggest that the isomer specific influence of CLA on glucose and lipid metabolism is genotype dependent and at least in part mediated by PPARγ.

**Trial registration:**

: ISRCTN91188075

## Introduction

The term CLA describes a group of 18-carbon fatty acids with 2 conjugated double bonds. They are formed as a result of enzymatic hydrogenation of linoleic acid in ruminants and found in lipids of tissue and milk. Of the various isomers, cis-9, trans-11 (c9t11) CLA is most predominant in food. In CLA preparations c9t11 CLA and trans-10, cis-12 (t10c12) CLA are the prevailing isomers. CLAs received considerable attention because of their effect on body weight, muscle mass and glucose homeostasis [1,2] in certain animal models. In humans evidence is less clear. CLA supplementation was shown to have significant favorable effects on body weight and fat mass [3,4] while results on other risk markers are still conflicting. The findings showed great variability, which may be partly explained by the length of the studies and the dosage, but also due to differences in the isomer mixtures used for supplementation. Additionally, the genetic background of individuals may have contributed to the variability of the results.

The PPARγ2 gene is a member of the nuclear receptor super family and is highly expressed in adipose tissue [5]. Indeed, the receptor plays a critical role in adipocyte differentiation and regulates insulin sensitivity by transcriptionally activating adipocyte-specific genes involved in insulin signaling, glucose uptake, fatty acid uptake and lipid-storage. A single nucleotide polymorphism (SNP) in the PPARγ2 results in a proline to alanine substitution and has been associated with lower risk for type 2 diabetes, higher insulin sensitivity, but paradoxically weight gain [6]. The polymorphism of PPARγ2 was shown to affect metabolic responses to dietary fat [7,8]. CLAs are natural PPAR-ligands [9]. Only one report is available which provides data on the CLA influence on gene expression in human adipose tissue [10].

To our knowledge this is the first human study using a microarray based genome wide gene expression screening to evaluate isomer specific CLA effects in human adipose tissue. In this study we further investigated whether CLA effects depend on the Pro12Ala polymorphism.

## Methods

### Subjects

38 subjects (45-68 years old) were recruited from a population based cohort (n = 1558) in Kiel (MICK, Metabolic Intervention Cohort Kiel) which has been previously described [11]. 15 male PPARγ2 Ala12Ala homozygous and 23 BMI-matched homozygous control subjects (PPARγ2 Pro12Pro) were included. Exclusion criteria were: metabolic or gastrointestinal diseases, drug therapy affecting gastrointestinal metabolism, and previously diagnosed diabetes according to WHO criteria (WHO, 2006). The intervention study was mono-centered and followed a randomized, placebo-controlled, double blind, cross over design. After written consent, 38 men randomly underwent four intervention periods, consuming soft gel capsules with either c9t11 CLA, t10c12 CLA, a commercially available mixture of both CLA isomers or placebo (linoleic acid from safflower oil) as free fatty acids; resulting in a total of 4.25 g/d fatty acids, containing either 3.40 g of the individual isomers or CLA mixture active substance or 3.23 g linoleic acid. Study participants completed four intervention periods lasting 28 days each; separated by wash-out phases lasting 42 days. The study protocol was approved by the local ethics committee of the Christian-Albrechts-Universität (Kiel, Germany).

### Sampling

After each intervention subcutaneous abdominal adipose tissue biopsies were performed under local anaesthesia by aspiration from the peri-umbilical area, through a Menghini-needle. Subcutaneous adipose tissue was immediately flash-frozen in liquid nitrogen for later analysis. BMI (body mass index), waist and hip circumference, blood pressure, plasma glucose and insulin, triacylglycerols, LDL-cholesterol HDL-cholesterol and HOMA-IR (homeostasis model assessment-insulin resistance) calculated as [(fasting insulin μU/ml × fasting glucose mg/dl)/405] were assessed after each intervention. Multiple ANOVA was used to compare effects of the 4 dietary interventions.

### RNA

RNA was extracted using Ambion kit according to the manufacturer's protocol for fatty tissue. RNA quality was verified by spectrophotometry (260:280 ratio) and by Bio analyzer (Agilent).

### Microarray

Global gene expression was analyzed with microarray technology using the Affymetrix Human Genome U133 2.0 Plus according to the manufacturer's protocol and the Gene Chip Set, covering more than 54 000 gene transcripts, corresponding to almost 22 000 genes. Expression values were calculated using the robust multi-array average algorithm [12] and were statistically analyzed for differential gene expression adjusting all P-values for false discovery-rate correction. The positive and negative controls met the expected values (data not shown). Wilcoxon test followed by Benjamini Hochberg Correction as post hoc test for multiple testing was used to analyse significant differences compared to intervention with linoleic acid, and Westfall and Young permutation was used for cut off calculation [13]. Pathway members were analysed by Ingenuity Pathway Analysis^® ^(Ingenuity Systems, ) software.

### RT-PCR

Purified RNA was reverse transcribed using Applied Biosystems High Capacity cDNA Reverse Transcription Kit according to manufacturer's recommendations. Realtime PCR was performed using an ABI Prism 7900 HT Sequence Detection System with TaqMan^® ^Low-Density Arrays, which is a 384-well micro-fluidic card pre-loaded with optimised probes and primers for selected genes. Relative quantification analysis was performed with ABI Prism SDS 2.1.1 software. The endogenous control TAF10 (TAF10 RNA polymerase II) was used to normalize the differences in amounts of the cDNAs added. Normalized delta (Δ) cycle threshold (CT) values were then subjected to evaluation of statistical significance (p < 0.05) of differential expression compared to control using the Wilcoxon test. Fold changes were calculated using the ΔΔCT method, whereby linoleic acid intervention values were used as reference (control).

## Results

37 out of 38 men completed the study. Data were based on 34 subjects; one dropped out because he fell ill and three subjects with PPARγ2 Pro12Pro SNP were excluded because they showed elevated fasting glucose levels according to the WHO criteria during most or all interventions. No significant changes in anthropometric characteristics and fasting values were detected after any intervention (Table 1).

**Table 1:** Anthropometric characteristics, fasting values of all subjects (n = 34) at the end of each intervention period.

**Preparation**	**linoleic acid**	**CLA MIX**	**cis-9,trans-11 CLA**	**trans-10,cis-12 CLA**	**P**
Weight (kg)	84.2 (± 2.0)	84.2 (± 2.0)	84.1 (± 2.0)	84.0 (± 2.0)	0.851
BMI (kg/m^2^)	26.1 (± 0.5)	26.1 (± 0.4)	26.0 (± 0.4)	26.0 (± 0.5)	0.900
Waist (cm)	102.1 (± 1.5)^a^	102.1 (± 1.6)^a^	102.3 (± 1.5) ^a^	101.2 (± 1.5) ^b^	**0.072**
Hip (cm)	104.8 (± 1.0)	104.8 (± 1.0)	104.8 (± 1.0)	104.0 (± 1.0)	0.824
Waist/hip ratio	0.97 (± 0.01)	0.97 (± 0.01)	0.98 (± 0.01)	0.97 (± 0.01)	0.645
Plasma glucose (mg/dl)	96.5 (± 1.4)	98.2 (± 1.5)	97.5 (± 1.5)	96.5 (± 1.4)	0.487
HOMA-IR	3.0(± 0.3)	3.2(± 0.2)	3.1(± 0.3)	2.9(± 0.2)	0.693
Triacylglycerols (mg/dl)	127.0 (± 10.1)	119.1 (± 8.9)	124.4 (± 10.8)	124.7 (± 10.6)	0.787
Total cholesterol (mg/dl)	223.9(± 7.3)	225.3(± 6.8)	223.6(± 7.2)	226.5(± 6.8)	0.783
LDL cholesterol (mg/dl)	56.3(± 2.6)	56.5(± 2.3)	56.7(± 2.5)	54.6(± 2.8)	0.268
HDL cholesterol (mg/dl)	145.4(± 6.0)	143.1(± 5.8)	145.7(± 6.3)	146.4(± 5.7)	0.772
SBP (mmHg)	127 (± 3)	127 (± 3)	128 (± 3)	126 (± 2)	0.901
DBP (mmHg)	80(± 2)	79 (± 2)	80 (± 2)	80 (± 2)	0.871

Values are expressed as mean ± SEM. CLA, conjugated linoleic acid; BMI, body mass index, DBP diastolic Blood pressure, SBP systolic blood pressure LDL low-density lipoprotein cholesterol, HDL high-density lipoprotein cholesterol, HOMA-IR; homeostasis model assessment-insulin resistance, intervention-dependent changes were compared using multiple ANOVA.

From five PPARγ2 Ala12Ala subjects and five BMI-matched Pro12Pro control subjects' microarray analyses were made after each intervention. The data have been deposited in NCBI's Gene Expression Omnibus and are accessible through GEO Series accession number GSE16615. The experiment showed the presence of approximately 12 000 gene transcripts in subcutaneous adipose tissue, belonging to about 1200 different genes. According to Wilcoxon test at p < 0.05, 310 genes were changed by the CLA preparations as compared to the control intervention. After Benjamini Hochberg Correction as post hoc test for multiple testing no intervention showed significant effects.

**Figure 1:**
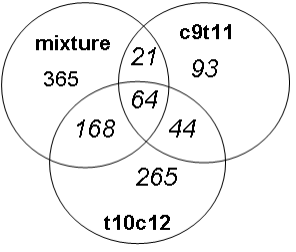
**Distribution of the gene transcripts (n = 1020) regulated more than +/- 1.5 fold after the various interventions compared to linoleic acid control as assessed by whole genome gene expression microarray U133 2.0 Plus GeneChip**. Intersections show genes regulated by more than one treatment.

According to Westfall and Young permutation the cut off for a significant difference in gene expression between CLA and control intervention was set at +/- 1.51 fold change. Among the genes exceeding this limit only those were further evaluated that were detected on all microarrays, showed annotated gene symbols and resulted in no hypothetical proteins. Following these criteria 1020 gene transcripts were differently expressed after CLA intervention compared to the control (Figure 1).

The expression of 42 genes was verified by realtime PCR after all interventions in all subjects. The criteria for gene selection were as follows: they showed a strong difference in the expression after one or more CLA interventions compared to the control in microarray analysis, they showed a different expression between the two genotype groups after an intervention, they are a member of a regulated pathway (pathway analysis with Ingenuity Pathway Analyses™) or they were previously described to be regulated by CLA.

Table 2 shows genes that were significantly regulated after CLA interventions (p < 0.05) or showed a strong tendency to be regulated (more than +/- 1.51 fold change and p ≤ 0.1).

**Table 2:** Fold change (median) of genes after 4 weeks intervention with a mixture of c9t11 and t10c12 CLA, c9t11 CLA, and t10c12 CLA compared to control (LA) according to realtime PCR verification.

	**MIX**			**C9T11 CLA**			**T10c12 CLA**			
**Gene symbol [NCBI RefSeq]**	**all**	**Pro**	**Ala**	**all**	**Pro**	**Ala**	**all**	**Pro**	**Ala**	

**Fatty acid transport and metabolism**										PPARγ dependency

ABCA9 [NM_080283.3]	-1.3*	-1.3*					-1.4*			n/a
LDLR [NM_000527.3]	1.2**	1.8**					1.5**	1.5**		↑[85]
CD36 [NM_001001547.1]				-1.2*		-1.6*	-1.3*		-1.2*	↑[65,86]
LIPE [NM_005357.2]			1.96†						1.8†	↑[23]
CA3 [NM_005181.2]							-2.0**	-2.0*		n/a
FASN [NM_004104.4]		1.5*					1.2*	1.4*		↑[87]
SCD [NM_005063.4]			1.3*				1.5*		1.9*	↑[34,88]
FADS1 [NM_013402.3]	1.6*	1.6*								n/a

**Transkription factors**										

PPARG [NM_138711.3]		-1.4**a	1.3 b							
NFAT5 [NM_138713.2]	-1.5*	-1.6*					-1.6*		-1.9†	n/a
CREB5 [NM_182899.3]							-1.5*		-1.9†	n/a
EBF1 [NM_024007.3]		-1.5†								↑[89]

**Adipokines**										

ADIPOQ [NM_004797.29]			1.6†						1.5†	↑[90,91]
LEP [NM_000230.2]		-1.2 a	1.7†b							↓[92]
NAMPT [NM_005746.2]									-1.6*	= [62]
THBS1 [NM_003246.2]				1.5**	1.3*	1.5*				↓[64] ↑[65]

**Insulin signaling**										

IGF1 [NM_001111284.1]		-1.5†					-1.2**	-1.2*		↓ [93]
SLC2A4 [NM_001042.2]	1.3*		1.5†							↑[94]
SORBS1 [NM_001034954.1]		-1.2*								↑[74]
PIK3R1 [NM_181523.1]		-1.6†								↑[95]

**Others**										

HPGD [NM_000860.3]	-1.3**		-1.4**						-1.6*	See text
API5 [NM_006595.2]							-1.3†		-1.5*	n/a
CD74 [NM_001025158.1]		1.2*								n/a
UCP2 [NM_003355.2]	1.14*								1.8†	↑[79]
IL6ST [NM_175767.1]	-1.3*						-1.5†		-1.8†	n/a

Comparison of CLA interventions and control (Wilcoxon test): *p < 0.05; **p < 0.001; † marks values with a tendency p ≤ 0.1 and more than +/- 1.5 fold change. Letters a and b indicate significant differences (p < 0.05) between effects in PPARγ2Pro12Pro (Pro; n = 23) and PPARγ2Ala12Ala (Ala; n = 11) carriers after the CLA mixture supplementation (Mann-Whitney U test).

## Discussion

To elucidate the mechanism of different effects exerted by individual CLA isomers and the mixture of both CLA isomers, a stepwise approach was applied. Following a hypothesis free approach by human genome wide gene expression screening, expression was verified by realtime PCR in those genes which showed measurable differences in expression compared to control intervention in the microarray experiment, which are members of relevant pathways or which were described in the literature as being regulated by CLA. Not all microarray results from a subgroup of five respectively ten persons could be verified for the whole study group by realtime PCR.

Microarray results indicated that single isomers and the CLA mixture of both isomers may modulate different genes. This was also confirmed by the results of realtime PCR (table 2). This may be due to the given structural differences between these isomers and due to the fact that more than one biochemical mechanism is supposedly involved in their specific effects.

### Fatty acid transport and metabolism

Microarray analyses showed that **ABCA9 **(ATP-binding cassette, sub-family A, member 9) was much less expressed after CLA interventions in the 10 selected subjects. Verification for all 34 study subjects with realtime PCR confirmed this result for the CLA mixture and the t10c12 CLA intervention and showed genotype specific effects.

Little is known about the physiological function of ABCA9 especially in adipose tissue. ABCA9 is a member of the ABCA6-like transport subclass, which is regulated by cholesterol in an opposite direction than ABCA1 [14]. In human macrophages, ABCA9 mRNA is induced during monocyte differentiation and inhibited by cholesterol influx in human macrophages, indicating that ABCA9 is a cholesterol-responsive gene [15]. Lower expression in adipose tissue after CLA mixture and t10c12 CLA intervention (Table 2) may be due to high influx of cholesterol in adipocytes, however, total plasma cholesterol did not change after any CLA treatment (table 1).

The **LDLR **(LDL receptor) regulates plasma LDL cholesterol levels. Yu-Poth et al. showed that CLA mixture induces LDLR expression in the HepG2 hepatic cell line [16]. Others found the strongest enhancing effect on LDLR expression in mouse liver after t10c12 CLA administration compared to linoleic acid, whereas c9t11 CLA showed no effect [17]. We also found a higher expression after CLA mixture and t10c12 CLA treatment (table 2). This effect was particularly found in Pro12Pro carriers. These results suggest that CLA may up-regulate LDLR gene expression to promote the clearance of LDL cholesterol from circulation. By that it may have inhibited ABCA9 expression (table 2). The observed effects of CLA on plasma lipids and particularly LDL-cholesterol in humans are conflicting. We found, however, no significant effects on plasma LDL or HDL cholesterol or triacylglycerol concentrations in our study (table 1).

**CD36**, another lipid transporter and scavenger receptor for oxidized LDL, was less expressed after c9t11 and t10c12 CLA treatment in all subjects. Lower expression of CD36 after t10c12 CLA incubation were also detected in mice 3T3-L1 cells [18]. Higher expression of LDLR and lower expressions levels of CD36 after t10c12 CLA treatment may be indicative of compensatory effects. Accordingly, LDLR-/- mice had higher CD36 expression [19]. Plasma glucose levels play an important role in the regulation of CD36 expression in adipose tissue [20]. Moreover, CD36 expression in subcutaneous adipose tissue was higher in type 2 diabetic subjects [21]. Ala12Ala carriers in particular showed significantly lower CD36 expression after the intervention with the single CLA isomers (table 2).

An explanation may be provided by an electrophoretic mobility shift assay experiment which showed that the PPARγ2 Ala-variant binds with lower affinity to an PPARγ-responsive element than the Pro-variant [22]. A reduced transcription of specific PPARγ target genes was reported for cells over-expressing the Ala-variant compared to Pro-variant over-expressing cells [22].

**LIPE **(hormone sensitive lipase) is a key enzyme in mobilization of fatty acids from intracellular lipid stores and is a PPARγ target gene in human liver cells [23]. It was 1.96 fold more expressed after CLA mixture (p = 0.06) and 1.8 fold more after t10c12 CLA (p = 0.09) treatment in the rare allele carriers. This does not support the findings from Nazare et al., who found a significantly lower expression of LIPE after a 14-week supplementation with CLA mixture in human adipose tissue [10], whereas lipoprotein lipase was not affected in their study. In contrast, in male Syrian Golden hamsters there was no effect on LIPE in adipose tissue after 3 weeks with a t10c12 CLA enriched diet [24].

**CA3 **(carbonic anhydrase 3) hydrates carbon dioxide to yield bicarbonate and carbohydrogen ion and has a variety of physiological functions. One is to provide bicarbonate ions necessary to convert acetyl-CoA into malonyl-CoA, the building block of fatty acids. CA3 is the most abundant protein of the rat adipose tissue and its expression decreased with obesity [25]. CA3 expression was decreased by leptin but increased by insulin in freshly isolated rat adipose cells, which suggests that the decrease in CA3 expression observed in obesity may be related to hyperleptinemia [26]. But in this study a significantly lower expression of CA3 was detected after t10c12 CLA compared to control intervention, while there was no change in leptin expression. These discrepant findings may be due to the known differences between humans and rodents [27]. The reduced expression of a key enzyme in fatty acid synthesis brought about by t10c12 CLA, however, fits the fat mass reduction observed in other studies [4].

**FASN **(fatty acid synthase) a multifunctional enzyme complex was significantly induced in carriers of the common allele after both CLA mixture and t10c12 CLA treatments and also in all subjects combined after t10c12 CLA treatment. FASN mRNA was up-regulated, leading to higher insulin concentrations in cultured human adipocytes [28]. Increased expression of FASN in adipose tissues was associated with excess fat accumulation and impaired insulin sensitivity [29].

**SCD **(stearoyl-CoA desaturase) is involved in fatty acid biosynthesis. The enzyme introduces a cis-double bond in the delta-9 position of certain saturated fatty acids and is the rate-limiting enzyme in the biosynthesis of monounsaturated fatty acids (MUFAs). Studies in animal models have shown that SCD is highly regulated by diet, but data in humans are limited [30]. As SCD is involved in the biosynthesis of MUFAs, namely palmitoleate (16:1) and oleate (18:1), the activity of SCD in adipocytes affects the fatty acid composition of cellular phospholipids, cholesterol esters and triacylglycerols [31]. In this study SCD expression was higher after t10c12 CLA supplementation in all subjects combined and for the rare PPARγ2 genotype both after CLA mixture and t10c12 CLA treatment. In contrast to this Brown et al. [32] found a lower expression in cultured human preadipocytes after incubation with t10c12 CLA compared to bovine serum albumin (BSA) control. Sjogren et al. showed that elevated SCD activity, measured as fatty acid desaturation index 18:1/18:0 in human adipose tissue, is closely coupled to the development of insulin resistance [33]. Another study however found no correlation between BMI, insulin sensitivity, triacylglycerol or plasma leptin levels and SCD expression in subcutaneous adipose tissue [34]. In the same study treatment with the PPARγ agonist pioglitazone increased the SCD expression and insulin sensitivity in subjects with impaired glucose tolerance [34]. SCD activity also increased the concentration of 16:1 and decreased the concentration of palmitate (16:0). Palmitate is a precursor of ceramides, which inhibit insulin-stimulated glucose transport and are involved in development of insulin resistance [35].

**FADS1 **(fatty acid desaturase 1) catalyzes endogenous synthesis of polyunsaturated fatty acids (PUFAs), including arachidonic acid and eicosanoids [36], which have, amongst others, proinflammatory and coagulatory effects. A higher FADS1 expression as observed in this study in Pro12Pro carriers and all subjects combined following the CLA mixture treatment may result in lower triacylglycerol and fasting insulin levels. Such a correlation was shown by Steffen et al. [37]. They found an inverse correlation between FADS1 activity measured as ratio of 20:4,n6/20:3,n6 and triacylglycerol as well as fasting insulin levels [37].

### Transcription factors

Pro12Pro carriers showed a significantly lower **PPARγ **gene expression after CLA mixture treatment, whereas there was no change in Ala12Ala carriers. Thus the effect on PPARγ gene expression differed significantly between the two genotypes. This is the first time that genotype specific effects of CLA on PPAR gene expression in human adipose tissue were detected. This might explain some of the different results obtained in different human studies investigating the influence of CLA.

Brown et al. showed that c9t11 CLA increased and t10c12 CLA decreased the mRNA expression of PPARγ in primary human adiopocytes in vitro [32]. Stratifying for PPARγ isoforms Chung et al. found a suppression of PPARγ and PPARγ2 at the mRNA and protein level in human cultured adipocytes after t10c12 CLA incubation [38]. Others, however, observed an increased expression of PPARγ in human adipose tissue after 14 weeks of a CLA mixture treatment [10]. As a key modulator in adipose tissue, PPARγ controls several genes in lipid and glucose metabolism and is involved in the regulation of several genes affected by CLA treatment (table 2) [23,34,49,62,64,65,74,79,85-95]. The role of CLA as PPARγ ligand is not fully understood until now.

Thiazolidinediones (TZDs) - strong synthetic PPARγ agonists used in diabetes therapy to improve insulin sensitivity - induce weight gain (reviewed in [39]) whereas CLAs, natural PPARγ ligands, caused moderate weight loss in humans [4,40] and in some studies reduced insulin sensitivity in an isomer specific way [41,42].

This points out that there are several differing regulatory mechanisms of CLA and TZDs which are partly PPARγ independent. These results spur the question if CLA or single isomers are either PPARγ agonist or antagonist. In cultured human primary adipocytes t10c12 CLA antagonized PPARγ activity ligand-dependently [32,43], whereas Miller et al. showed that t10c12 CLA is a PPARγ agonist and that treatment of 3T3-L1 cells with t10c12 CLA decreases adiponectin through PPARγ-dependent and PPARγ-independent mechanisms [44].

Very recent studies on PPARγ-DNA binding activity in human umbilical vein endothelial cells after treatment with the individual CLA isomers showed that the concentration determines whether CLA isomers exert inhibitory or a stimulatory effects in inflammatory and atherogenic processes, and that microenvironments also influence CLA effects [45]. The tissue dependent differences of action were also evident in mice; t10c12 CLA induced loss of adipose tissue but at the same time decreased fatty acid oxidation and increased fatty acid synthesis in liver [46].

Thus CLA effects depend on concentration, tissue, species, isomers, and once they are potential PPARγ ligands, maybe also on PPARγ polymorphisms.

**NFAT5 **(nuclear factor of activated T-cells 5) refers to the Rel family of transcription factors, which also comprises NFκB and NFATc. The exact pathways for NFAT5 regulation are largely unknown. NFAT5 expression was reduced following CLA mixture and t10c12 CLA treatment. High glucose concentrations increased binding activity of NFAT5 to osmotic response elements in both peripheral blood mononuclear cells and human mesangial cells, and silencing of NFAT5 reduced expression of aldose reductase (ADR) [47]. ADR is the first and rate-limiting enzyme of the polyol pathway where unused glucose is metabolized to sorbitol. ADR expression was associated with rapid development of diabetic microangiopathy in Japanese Type 2 diabetic patients [48]. However, in our microarray experiment ADR gene expression was not affected by treatments (data not shown).

**CREB5 **(cAMP responsive element binding protein 5) is another transcription factor which was less expressed after t10c12 CLA compared to control treatment in this study. Little is known about the function of CREB5. It is a member of the activating transcription factor 2 cAMP-binding protein family and is activated by a variety of kinases including protein kinase A and is involved in tumorigenesis of endocrine tissues and different forms of leukemia [49]. CREB5 was more expressed in omental adipose tissue biopsies of women with metabolic syndrome than in those of healthy women [50]. The lower expression levels after t10c12 CLA intervention is rated as a positive effect on metabolism.

The transcription factor **EBF1 **(early B-cell factor 1) is up-regulated during adipocyte differentiation. In mouse adipose tissue Granlund et al. found no change after t10c12 CLA compared to BSA incubation [51]. This is in agreement with our realtime PCR results. We detected only a slight down regulation in the common genotype after CLA mixture treatment.

### Adipokines

**LEP **(leptin) reduces fat depots via mediating the degradation of fatty acids by inducing uncoupling protein 2 (UCP2) and carnitine palmitoyltransferase-1 (CPT1), key enzymes of β- oxidation [52], by decreasing acetyl-CoA carboxylase (ACC) [53] and by decreasing food intake [54]. Leptin, however, induces mitochondrial superoxide production and MCP1 (monocyte chemotactic protein 1) expression in endothelial cells [53], endothelial dysfunction [55] and CRP (C-reactive protein) expression [56]. Effects of CLA on leptin levels are conflicting. In Wistar rats CLA supplementation decreased serum leptin levels [57], while no differences were found after CLA supplementation in humans [10].

In this study none of the CLA supplements as compared to linoleic acid control affected gene expression, which confirms our microarray results. There was, however, a genotype specific difference after CLA mixture treatment as assessed by realtime PCR (Table 2); rare allele carriers had higher expression levels than PPARγ2 Pro12Pro carriers. Little is known about the functional impact of Pro12Ala polymorphism on leptin levels in humans. The lower leptin gene expression after CLA mixture treatment in subjects with the common genotype are in line with lower expression levels of PPARγ in this genotype. Same tendency was found for plasma leptin concentrations in this study (unpublished data).

Accordingly, **ADIPOQ **(adiponectin) gene expression, which depends on PPAR regulation, tended to be higher in rare allele carriers after CLA mixture and t10c12 CLA treatment (Table 2). While in young Finnish men Ala12Ala genotype of PPARγ2 was associated with elevated adiponectin level [58], the Ala12Ala allele was associated with lower serum adiponectin concentrations in Japanese men [59]. The same tendency was found in the MICK cohort [7]. These differing effects of PPARγ2 may be due to different dietary backgrounds. In the murine 3T3-L1 cell line, t10c12 CLA compared to a non-stimulated control decreased adiponectin levels by PPARγ-dependent and PPARγ-independent mechanisms, whereas c9t11 CLA showed no effect [44]. In mice higher adiponectin levels were detected after treatment with pomegranate seed oil, a rich source of c9t11 CLA [60].

In rare allele carriers **NAMPT **(visfatin) was significantly less expressed after t10c12 CLA supplementation compared to control. Plasma visfatin levels were increased in obese and insulin-resistant humans [61], and plasma levels and adipocyte gene expression were not affected by PPARγ ligands [62,63].

Gene expression of the anti-angiogenic adipokine **THBS1 **(thrombospondin 1) was significantly higher after c9t11 CLA intervention according to our microarray and realtime PCR results. This adipokine is highly expressed in subcutaneous adipose tissue of obese and insulin-resistant subjects and is decreased by pioglitazone [64]. On the other hand, in endothelial cells THBS1 efficacy was improved by troglitazone through a PPARγ and CD36 dependent mechanism [65], which could result in an anti-angiogenic action.

### Insulin signaling

**IGF1 **(insulin-like growth factor 1) was less expressed after t10c12 CLA supplementation in PPARγ2 Pro12 Pro carriers and all subjects combined, and in PPARγ2 Pro12 Pro carriers the expression was also slightly down regulated after CLA mixture treatment. These results are in line with findings in human colon cancer cells incubated with CLA mixture [66]. In adipose tissue IGF1 regulates adipogenesis, stimulates lipid oxidation, reduces protein oxidation, and enhances insulin sensitivity in humans [67]. Low serum IGF1 concentrations in humans were associated with lower visceral but not with lower subcutaneous or total fat mass [68] and IGF1 and blood glucose levels were inversely correlated in obesity before and during energy restriction [69]. These results suggest that lower expression levels of IGF1 might result in lower insulin sensitivity after t10c12 CLA treatment, which is in line with the lower CA3 and higher FASN expression observed in our study (Table 2).

The expression of GLUT4 (**SLC2A4**; solute carrier family 2, member 4), another key PPARγ2 target gene, was enhanced after CLA mixture supplementation compared to control, suggesting an improved insulin sensitivity. The effect was somewhat (not significantly) higher in rare allele carriers alone. In mice a c9t11 CLA-enriched diet increased adipose tissue plasma membrane GLUT4 and insulin receptor expression compared with the control linoleic acid-enriched diet [70]. While c9t11 CLA had no effect on GLUT4 expression in human preadipocytes [32], t10c12 CLA repressed GLUT4 in 3T3-L1 cells [18]. This may explain the induction of insulin resistance by the t10c12 CLA isomer found in some but not all human trials [71,72]. In our trial HOMA-IR did, however, not deteriorate during t10c12 CLA administration compared to the control (Table 1).

After CLA mixture intervention **SORBS1 **(sorbin and SH3 domain containing 1) gene expression was lower in microarray experiment; but realtime PCR confirmed this result only for the common PPARγ2 genotype. SORBS1 acts as an adaptor protein in the insulin-signaling pathway. It is partly dissociated from the insulin receptor complex and bound to c-Abl protein upon insulin stimulation [73]. TZDs, specific synthetic ligands of PPARγ, can increase the expression of SORBS1 in adipose tissue [74]. Since SORBS1 is a PPARγ target gene, this result is in line with the lower expression of PPARγ after CLA mixture intervention in common allele carriers.

By alternative splicing of the **PIK3R1 **gene (phosphoinositide-3-kinase, regulatory subunit 1 alpha), the phosphatidylinositol 3-kinase (PI3K) regulatory subunit isoforms p85α, p55α and p50α are generated [75]. PI3K has been shown to regulate most of the insulin action [76]. Thus the lower gene expression level of PIK3R1 gene found after CLA mixture supplementation in the common allele carriers may have counteracted GLUT4 induction. Taken together the findings of counteracting actions in the insulin signaling pathway (IGF1, GLUT4, SORBS1, PIK3R1) (Table 2) may explain why CLA did not alter insulin sensitivity in our human trial (Table 1).

### Other regulated genes

**HPGD **(hydroxyprostaglandin dehydrogenase 15-(NAD)) catalyzes the oxidation reaction prostaglandin E 2 (PGE2) to inactive 15-keto-PGE2 [77], which can act as a ligand of PPARγ to increase the recruitment of coactivators. Overexpression of HPGD increased PGE2-dependent activation of PPARγ in 3T3-L1 adipocytes [77]. In the present study there were lower expression levels after CLA mixture supplementation in the rare allele carriers and in all study subjects combined (Table 2). The Ala12Ala subgroup showed also significantly lower expression after t10c12 CLA treatment. Thus the repression of HPGD by CLA may have weakened PPARγ mediated effects in this study.

**IL6ST **(interleukin 6 signal transducer) is shared by many cytokines, including pro-inflammatory interleukin 6 (IL6). IL6 was shown to induce the plasminogen activator inhibitor-1 (PAI1) production in cultured human preadipocytes [78]. Rega et al. speculated that IL6, by up-regulating PAI1 in adipose tissue, might contribute to the increased cardiovascular risk of patients with obesity and insulin resistance syndrome due to the auto- or paracrine actions in human adipose tissue [78]. Thus the repression of IL6ST after CLA mixture and t10c12 CLA intervention might indicate counteraction to systemic inflammation.

**UCP2**, a mitochondrial uncoupling protein that decreases ATP synthesis coupled to energetic substrates oxidation expression, was significantly more expressed after CLA mixture intervention compared to the control. These results are consistent with those of Ryder et al. who found higher expression of UCP2 in mice adipose tissue after CLA mixture supplementation [2]. They also detected an up-regulation after c9t11 CLA treatment. TZDs can stimulate the expression of UCP2 gene, probably via PPAR gamma [79]. It was shown that regulation of adiponectin gene expression is also mediated specifically by mitochondrial UCP2 levels [80]. Higher levels of UCP2 were detected after t10c12 CLA in the PPARγ2 rare allele carriers (Table 2) and are in line with the fact that adiponectin levels were also higher in these subjects (data not shown).

**CD74 **showed a higher expression level after CLA mixture intervention (Table 2). Nothing is known about the function of CD74 in adipose tissue. In lymphocytes CD74 introduction leads to NFκB activation [81].

**API5 **(apoptosis inhibitor 5) gene expression was significant lower in rare allele carriers after t10c12 CLA treatment (Table 2). API5 was shown to suppress apoptosis [82]. Suppression of API5, therefore, might mediate the antitumor effects of CLA found in several studies [83].

## Conclusion

CLA intake was shown to reduce BMI and body fat in overweight subjects in a number of studies [4]. Up to now, several human studies have been published but the effects on the risk markers of atherosclerosis and on traits of metabolic syndrome were rather inconsistent. Only few data are available on the effect of individual CLA isomers. This study provides novel information about the effects exerted by single CLA isomer intake on several genes as assessed by genome-wide gene expressions in human adipose tissue, prompting further insights in the mechanism of CLA action. Furthermore this is the first study in humans which focused on gene-nutrient interactions with respect to the PPARγ2 Pro12Ala SNP and CLA isomers.

Only two genes showed significant effects after c9t11 CLA intervention. This confirms the microarray results from Murphy et al [84], where there was no significant alteration in gene expression after the incubation of CaCo2 cells with c9t11 CLA, but plenty of effects after t10c12 CLA stimulation compared to linoleic acid control.

In summary the intervention with CLA mixture had the strongest effect on the key enzyme of PUFA synthesis, FADS1, which may indicate anti-inflammatory and anti-arteriosclerotic effects. t10c12 CLA showed regulatory effects on various genes: lower expression levels of CA3 and IGF1 after t10c12 CLA treatment in the whole group could be a sign for a beginning insulin resistance. On the other hand, higher expression levels of SCD, a key enzyme of MUFA synthesis, lower expression of visfatin, the tendency towards higher levels of adiponectin and the reduction in CREB5 gene expression in rare allele carriers indicate beneficial effects of t10c12 CLA for this genotype.

The CLA mixture treatment caused significant genotype specific differences in expression of PPARγ and the PPAR dependent leptin gene. The fact that the CLA effect on expression of PPARγ and leptin genes depends on the PPARγ2 Pro12Ala polymorphism suggests that PPARγ2 contributes to regulatory effects of CLA. The results of the PPARγ dependent genes, however, do not show a uniform pattern and interfering factors like HPGD further diversified response to dietary intervention, reflecting the complexity of interacting regulators.

## Competing interests

The authors JH, DR, RH, UH, MP, AA, CL, PW and SS declare that they have no competing interests.

DB is employee of Cognis GmbH. JS received research support from Cognis GmbH for this and several other studies, JS gave a talk on CLA paid for by Cognis GmbH

## Authors' contributions

JH, DR, UH, MP, CL, PW and JS were responsible for the conception and design of the study, JH, UH, DR, CL, and PW for the conduct of the study; JH, DR, RR, MP, SS and PW for data acquisition; JH, RH and AA for statistical analysis; JH, DR, UH, RH and JS for data interpretation; JH and JS for drafting the manuscript; DR, UH, DB, CL, AA, MP and JS for critical revision; JS and DB for handling funding and supervision.
